# Emergence of Marburg virus disease in Ethiopia: Implications for public health preparedness and its impact on Ethiopia’s health system

**DOI:** 10.1371/journal.pntd.0014394

**Published:** 2026-06-05

**Authors:** Sibhatu Biadgilign, Mesfin Fransua, Anteneh Eshetu, Abdu Bedru

**Affiliations:** 1 Independent‌‌ Public Health Analyst and Research Consultant, Addis Ababa, Ethiopia; 2 Department of Internal Medicine, Division of Infectious Diseases, Morehouse School of Medicine, Atlanta, Georgia, United States of America; 3 Department of Internal Medicine, Division of Infectious Diseases, College of Health Sciences, Addis Ababa University, Addis Ababa, Ethiopia; 4 School of Medicine, College of Medicine and Health Science, Wolkite University, Wolkite, Ethiopia; Public Health Agency of Canada, CANADA

## Abstract

**Background:**

Marburg virus disease (MVD) is a rare but highly lethal viral hemorrhagic fever (VHF) caused by Marburg virus (MARV) and Ravn virus (RAVV). While MVD has historically been limited to specific areas of sub-Saharan Africa, recent outbreaks in previously unaffected countries indicate an expanding ecological and epidemiological risk. In November 2025, Ethiopia confirmed its first-ever MVD outbreak, constituting a significant national and regional public health emergency.

**Methods:**

This narrative review synthesizes publicly available epidemiological data, government situation reports, and official communications from the Ethiopian Ministry of Health (MoH), Ethiopian Public Health Institute (EPHI), Africa Centres for Disease Control and Prevention (Africa CDC), and the World Health Organization (WHO).

**Results:**

The outbreak was initially detected in Jinka town, South Ethiopia Region, an area bordering Kenya and South Sudan. As of 25 January 2026, more than 3,800 diagnostic tests were conducted, leading to a total of 19 cases comprising 14 confirmed (including nine deaths) and five probable (all deaths) and five recoveries from MVD in the country’s South Ethiopia and Sidama regions, which were reported. A total of 857 contacts listed for monitoring all had completed their 21-day follow-up as of 25 January 2026. Ethiopia’s response included rapid notification, laboratory confirmation, activation of incident management systems, deployment of mobile high-biosafety laboratories, establishment of isolation and treatment centers, and issuance of national clinical management guidelines. International partners provided technical, financial, and logistical support. However, the outbreak exposed ongoing challenges, including health system fragility, workforce shortages, misinformation, funding limitations, and heightened cross-border transmission risk.

**Conclusion:**

The emergence of MVD in Ethiopia represents a pivotal moment for national and regional health security. Sustained containment will require strengthened surveillance, community engagement, cross-border collaboration, and integrated One Health approaches. Long-term investment in resilient health systems and coordinated regional preparedness is essential to prevent future spillover events and protect vulnerable populations.

## Background

Marburg‌‌ virus (MARV), a highly pathogenic single-stranded RNA virus that belongs to the Filoviridae family, is the cause of Marburg virus disease (MVD). MVD, formerly known as Marburg hemorrhagic fever, is a rare but fatal illness. It causes severe hemorrhagic fever clinically similar to Ebola virus disease [1]. Zoonotic spillover from fruit bat reservoirs (notably *Rousettus aegyptiacus*) followed by person-to-person transmission by direct contact with bodily fluids, is the established transmission pathway [1]. According to the World Health Organization (WHO), the average fatality rate is 50%, although the rates range from 24% to 88% in past outbreaks, depending on virus strain and case management [2]. The WHO first recognized MARV during an outbreak in 1967 that occurred simultaneously in Germany and Serbia (then part of Yugoslavia) [2]. MARV was unknown prior to the outbreaks in Germany and Yugoslavia. Recent and previous outbreaks and sporadic cases of MVD in regions of sub-Saharan Africa without prior record reported in Ghana, Guinea, Kenya, South Africa, Angola, Tanzania, the Democratic Republic of the Congo (DRC), Equatorial Guinea, Uganda, and Rwanda [1,3] challenge the current understanding of areas at-risk for filovirus spillover [3,4].

## Epidemiology, outbreak notification, and diagnostics of MVD in Ethiopia

The English Reporter, which is a widely read national newspaper in Ethiopia, known for its broad reach and influence among diverse audiences in its November 22, 2025, publication described how the unfolding outbreak has deeply unsettled residents of southern Ethiopia. The article reported that fear and shock quietly took hold in the community after, within just a few days, two health workers, a church leader, and a police officer lost their lives. Each of these cases was reportedly connected through a chain of exposure that spread across Jinka General Hospital and into the surrounding communities [5]. On November 12, the Ministry of Health (MoH) announced suspected viral hemorrhagic fever (VHF) in Jinka town, South Ethiopia Regional State, Ethiopia and on November 14, 2025, the MoH confirmed that the country was facing its first recorded MVD outbreak [6]. Geographically, the outbreak has been concentrated in Jinka town, a key urban center in the South Ethiopia Region, the first of its kind in the country, which is situated near Ethiopia’s border with South Sudan and Kenya. Reports indicated that the preliminary genomic analysis suggested that the detected virus strain showed similarities to those that had been identified in previous outbreaks in other East African countries (consistent with regional lineage diversity), though detailed sequence data have not been widely released in the public domain [3,6]. Following laboratory confirmation, the ministry formally notified Africa Centres for Disease Control and Prevention (Africa CDC) and the WHO after MARV was detected in samples from a cluster of suspected VHF cases. The MVD outbreak was declared over by the MoH of Ethiopia on 26 January 2026. This announcement was made following the official declaration that marked the completion of 42 consecutive days (equivalent to two incubation periods of 21 days) following the death of the last lab-confirmed case of MVD. The person died on 14 December 2025 and was buried safely and with dignity in accordance with WHO guidelines [2]. As of 25 January 2026, more than 3,800 diagnostic tests were conducted, leading to a total of 19 cases comprising 14 confirmed cases including nine deaths (Case Fatality Rate (CFR) of 64.3%) and five probable cases (all of whom had died) and five recoveries from MVD from Jinka, Malle and Dasench woredas in South Ethiopia Region and Hawassa in Sidama Region were reported [2]. A total of 857 contacts listed for monitoring all had completed their 21-day follow-up as of 25 January 2026. One of the recently identified cases in Hawassa town, Sidama Region tested positive and was traced back to Jinka town, and is closely linked to the index case, indicating a clear epidemiological connection in the transmission chain and cycle [2].

Table 1 below presents a chronological overview of key outbreak detection and response events that occurred between November 2025 and January 2026, outlining how the situation unfolded over time and the corresponding public health actions taken.

**Table 1:** Timeline of outbreak detection and response events (Nov 2025–Jan 2026).

Date/Period	Event	Details/Response Actions
11 November 2025	Initial alert of suspected cases	Health facilities in southern Ethiopia reported a cluster of patients with severe symptoms consistent with VHF, including high fever, bleeding manifestations, and acute weakness.
12–13 November 2025	Sample collection and rapid investigation	The MoH deployed rapid response teams. Clinical samples were collected from suspected VHF cases and prepared for laboratory testing. Active case finding and contact listing were initiated in affected communities.
14 November 2025	Laboratory confirmation and outbreak declaration	Samples were transported to the national reference laboratory. Laboratory testing confirmed MVD. The MoH officially declared an outbreak and notified partners, including WHO. Emergency surveillance systems were strengthened, and WHO deployed support teams.
16 November 2025	Activation of national emergency response	The Public Health Emergency Operations Center (PHEOC) was activated at full capacity. Rapid response teams were deployed to the affected areas. Isolation and case management protocols were reinforced.
17 November 2025	Early outbreak update	MoH reported 17 suspected cases, including three deaths among confirmed cases and three deaths among suspected cases.
19 November 2025	Situation report update	MoH began issuing daily situation reports. Four confirmed cases were reported among 28 suspected cases, with three confirmed deaths to date.
17–20 November 2025	Scale-up of field response	Expanded contact tracing and active surveillance were implemented. Infection prevention and control(IPC) measures were strengthened. Health workers trained on case management and personal protective equipment (PPE) use. Community awareness campaigns were launched.
21 November 2025	Epidemiological update	Confirmed cases increased to eight, with four deaths (CFR: 50%). 46 samples tested. WHO reported 206 contacts under follow-up.
24 November 2025	Case progression update	Confirmed cases rose to 10, with five deaths. Five patients were received treatment.
27 November 2025	Geographic spread and guideline release	Confirmed cases increased to 12, with seven deaths. First case detected in Hawassa in a traveler from Jinka. National Marburg case management guideline released.
21–30 November 2025	Coordination and containment strengthening	Multi-sectoral coordination intensified between MoH, WHO, and partners. A National Taskforce was established at the MoH to provide strategic guidance, make key decisions, and mobilize the necessary resources. Emergency Operations Centres were activated at both national and regional levels, and incident management structures were put in place to coordinate and oversee the response activities. Logistics, surveillance tools, and laboratory capacity were scaled up. In addition, a costed three-month national response plan was developed and officially launched to guide the overall emergency response efforts.
02 December 2025	Case update	Confirmed cases increased to 13, with eight deaths reported.
Early December 2025	Transmission monitoring phase	No significant new clusters reported. Contact follow-up continued through incubation period.
08 December 2025	Vaccine trial initiation	Phase 2 open-label Chimpanzee Adenovirus Type 3 (cAd3)-Marburg vaccine trial initiated in affected areas.
12 December 2025	Epidemiological update	14 confirmed cases and eight deaths reported.
Mid–Late December 2025	Outbreak control phase	Enhanced surveillance continued, while response teams evaluated transmission status and health facilities remained prepared and on alert.
06 January 2026	No new cases milestone	No new cases reported for 21 consecutive days.
26 January 2026	Outbreak declared over	Outbreak officially declared ended following epidemiological verification.
January 2026	Post-outbreak recovery phase	Post-outbreak assessment conducted including surveillance review, response evaluation, and survivor follow-up with psychosocial support.

## Government public health response for the outbreak

Ethiopian health authorities, together with several news agencies, reported that at least three deaths had occurred after laboratory tests confirmed suspected cases of VHF. In response, active public health measures were implemented, including community-wide screening, case isolation, treatment, contact tracing, and public awareness campaigns, all of which were established to help curb the spread of the MARV [3].

Parallel to this, the Ethiopia’s MoH, EPHI, and the WHO regional AFRO, and Africa CDC announced coordinated response activities that include: activation of incident management and rapid response teams; case identification, isolation, and clinical management with strict IPC measures in health facilities around Jinka Hospital and surrounding health centers, intensive contact tracing and risk-based monitoring of contacts; deployment of Africa CDC and WHO technical teams and provision of PPE, infection-prevention supplies, and outbreak-support tents [3]. More importantly, the MoH took a proactive role by facilitating the development and issuing the first edition of the Ethiopian National Guidelines for the case management of MVD, as well as outlining standards for facility readiness to effectively respond to potential cases [7].

In the context and framework of the WHO Joint External Evaluation (JEE) tool used to assess a country’s ability to prevent, detect, and respond to public health threats under the International Health Regulations (IHR, 2005) the codes/designations “D2” and “D2.3” refer to specific elements within the Detect core capacity, particularly under the surveillance technical area. The D2 indicator relates to real-time surveillance systems, which are designed to ensure the timely detection of potential public health events. In this structure, the “D” category represents the broader Detect function, while D2 specifically focuses on surveillance as a key technical area for early identification of health threats. The sub-indicator D2.3 refers to the third component under the surveillance domain. This element has traditionally focused on the analysis, interpretation, and reporting of surveillance data, ensuring that collected information is processed effectively and shared with relevant stakeholders to support timely public health action [8]. According to the WHO JEE (IHR 2005) mission report for Ethiopia (18–22 September 2023), the country demonstrated strong performance in the surveillance technical area. In particular, both early warning surveillance (D2.1) and data analysis and information sharing (D2.3) were assessed as high-performing components, each receiving a score of 4 out of 5, reflecting a well-functioning surveillance and information management system during the evaluation period [8]. In fact, the MoH quickly recognized the possibility of a VHF and promptly shared concerns with WHO and Africa CDC. This eventually led to rapid response including resource mobilization. In addition, Africa CDC has been supporting the capacity building at the MoH in VHF detection and sequencing as a result of the JEE. Testing capacity has been essential in confirming the MVD outbreak and ensuring an effective response. During its press briefing session on November 26, 2025, the government emphasized that it has intensified its response efforts.

An incident management system has been activated at both federal and regional levels to coordinate the outbreak response. The installation and deployment of an ambulatory/mobile laboratory service in Jinka for timely confirmation, having a high biosafety, was sent to South Ethiopia, Jinka town as part of the rapid response measures to the recent MVD outbreak in the region. The aim was to enhance diagnostic capacity locally, release laboratory results immediately, and collect more samples. To support safe and effective control of the outbreak, EPHI developed specific biosafety guidance to govern the use of diagnostic methodologies. For initial detection using reverse transcription polymerase chain reaction (RT-PCR), as well as for other high-risk laboratory procedures, testing should be conducted in Biosafety Level 3 (BSL-3) or enhanced BSL-3 (BSL-3+) containment facilities. Procedures involving viral culture, along with any laboratory activities that are likely to generate aerosols, must be performed under Biosafety Level 4 (BSL-4) containment to ensure the highest level of protection for laboratory personnel and the surrounding environment. Isolation centers have been established in affected areas with two hospitals designated as treatment centers, with dedicated health workers deployed to manage cases, trained personnel have been deployed, and essential medical supplies and equipment are being mobilized to ensure comprehensive care for patients. Furthermore, Ethiopia is collaborating with countries that have previously experienced Marburg outbreaks to exchange technical expertise, learn from their lessons, and secure access to treatments and vaccines that have shown promising outcomes. Notably, Remdesivir has been administered to frontline healthcare workers and individuals who were exposed, as part of efforts to improve clinical outcomes and ensure treatment availability within the country. Parallel to this, in regions where no cases have been detected, preparedness activities remain ongoing. Screening procedures have been strengthened at airports, border crossings, and other points of entry and exit locations, reinforcing efforts to prevent the spread of the virus into new areas.

## International partners movement and support

WHO Director-General Tedros Adhanom Ghebreyesus stated on social media, via his X (formerly Twitter) page, that his office had been collaborating closely with the MoH, EPHI, and regional health authorities as part of the rapid response efforts [9]. Following that, the WHO has released USD 300,000 from its contingency fund for emergencies in response. The organization stated that the funds would provide immediate support to the national response. The Africa CDC and WHO publicly commended Ethiopia for its rapid detection and transparent reporting, and both agencies provided technical support and strengthened surveillance efforts to help limit the spread. At the same time, the country intensified its response to halt the transmission of the virus and bring the outbreak to an end [10]. Neighboring countries—particularly South Sudan—have issued advisories and heightened surveillance at border areas because of cross-border population movement and regional health system vulnerabilities. In addition to the agencies already engaged, the Intergovernmental Authority on Development (IGAD) has warned that the ongoing MVD outbreak in Ethiopia could spread to other member states. In response to this risk, IGAD, in collaboration with the WHO, convened an emergency high-level meeting under the Regional Preparedness for Pandemic Response (PREPARE) initiative. Delegates from Ethiopia, Kenya, Uganda, and Djibouti attended the session, emphasizing that the outbreak’s close proximity to the borders with Kenya and South Sudan presents a serious regional public health threat. WHO has also deployed an 11-member multidisciplinary team of technical officers with extensive experience in responding to VHF outbreaks, along with essential medical supplies and equipment [3]. According to the WHO, this team strengthened disease surveillance, case investigation, laboratory testing, and community engagement activities.

African regional and multilateral partners played a critical role in the response, including the Africa CDC, IGAD, EPHI, and the Armauer Hansen Research Institute (AHRI). In addition, several United Nations agencies—most notably the WHO and United Nations Children’s Fund (UNICEF) were actively involved in providing technical guidance and operational support. Non-African bilateral and multilateral partners also made substantial contributions. These included the United States Centers for Disease Control and Prevention (US CDC), the European Union (EU), Germany through Deutsche Gesellschaft für Internationale Zusammenarbeit (GIZ) and the Robert Koch Institute, and the United Kingdom via the UK Health Security Agency (UKHSA). These partners provided specialized expertise in epidemiology, laboratory systems strengthening, outbreak analytics, and overall response coordination. Furthermore, international non-governmental organizations (INGOs), such as Médecins Sans Frontières (MSF) and the International Federation of Red Cross and Red Crescent Societies (IFRC), supported clinical case management and IPC in health facilities. They also mobilized community volunteers to strengthen surveillance, contact tracing and follow-up, and community engagement efforts, while ensuring the implementation of safe and dignified burial practices.

## Regional risk, challenges, and cross-border implications

While these are all laudable efforts, several factors raise concerns about the potential for wider regional spread and operational challenges. First, border proximity and population movement were identified as key challenges. Jinka’s location near international borders, combined with its role as the epicenter of the ongoing MVD outbreak, posed a significant cross-border public health risk. Its closeness and road connections to Kenya and South Sudan increased the likelihood of cross-border transmission, particularly into areas where surveillance systems were weaker, and health services remained limited. Second, uneven health system capacity across neighboring zones and countries contributed to delays in outbreak detection and hindered effective case management and IPC measures. Fragile health infrastructures in these areas made coordinated response efforts more challenging. Finally, the presence of zoonotic reservoirs and ecological risks added another layer of complexity. Rousettus bats were widely distributed in the region, and human–bat interactions such as during mining, cave exploration, or fruit harvesting remained common. These ongoing interfaces created the potential for repeated spillover events, highlighting the urgent need for strengthened One Health surveillance [6]. In considering regional patterns of viral hemorrhagic fever outbreaks, it is important to recognize that neighboring countries have different levels of experience and capacity in managing these emergencies. For example, Kenya, Sudan, and Uganda as well as multiple African countries, including Angola, Rwanda, and South Africa have previously experienced outbreaks of viral hemorrhagic diseases including MVD and related infections and have developed practical experience in outbreak investigation, surveillance, and coordinated response efforts. Strengthening transboundary surveillance efforts should consider such differences and encourage experience and information sharing and collaborative work.

Ethiopia’s health system was expected to face significant strain during the outbreak, particularly in remote and underserved areas. In these settings, providing adequate isolation, effective case management, and systematic contact tracing was already challenging due to limited infrastructure and ongoing workforce shortages. Rural health facilities often operated with constrained diagnostic capacity, inconsistent supply chains, and weak referral systems. Together, these limitations reduced their ability to detect and respond effectively to emerging infectious disease threats [11]. Furthermore, unlike previous public health emergencies such as COVID-19, cholera, or measles, this outbreak unfolded at a time when the health sector was already grappling with an increasingly fragile financing environment. Declining donor contributions had led to substantial funding gaps and constrained access to essential medical supplies and commodities [11–13]. This challenge was especially pronounced in eight of the world’s 26 poorest countries—South Sudan, Somalia, Democratic Republic of the Congo, Liberia, Afghanistan, Sudan, Uganda, and Ethiopia where more than one-fifth of external health assistance had been provided by the United States Agency for International Development (USAID) [14]. In addition, one of the major challenges in responding to the MVD outbreak in Ethiopia had been the widespread circulation of misinformation and rumors regarding the number of reported cases. Conflicting accounts from government authorities, media outlets, and particularly social media platforms had created confusion and heightened public anxiety. These inconsistencies made it increasingly difficult for health officials to communicate clear and reliable information, while also making it harder for the public to distinguish verified facts from speculation. Moreover, the circulation of controversial or unverified case figures online had not only intensified fear but also undermined effective outbreak response efforts, weakened community engagement, and eroded trust in public health interventions.

## Treatment and vaccine candidate

There are no approved/fully licensed antivirals(investigational and emergency use), monoclonal antibodies, or vaccines specifically for MVD. So far, no therapeutic or vaccine has received full regulatory approval for MARV anywhere globally, including in the African region. Clinical care remains primarily supportive, including adequate rest and hydration, close monitoring and management of oxygen saturation and blood pressure, prompt treatments of any secondary infections, and symptom care [15]. Given the absence of specific treatments, controlling the transmission of the virus and implementing strict infection-prevention measures remain crucial to limiting the spread of the disease and protecting public health. On November 15, 2025, the Government of Ethiopia, through the Ethiopian Food and Drug Authority (EFDA), has developed a guideline to support the emergency use authorization of vaccines and therapeutic products for the prevention and treatment of MVD. This guideline, titled “Guideline for Emergency Use Authorization of Vaccines and Therapeutics for Marburg Virus Disease,” with Document Number: EFDA/GDL/057 provides a regulatory framework to ensure that safe, effective, and quality-assured medical products can be made available rapidly during public health emergencies [16]. Nevertheless, important progress is being made through the use of investigational vaccine candidates. As of 4 December 2025, the Sabin Vaccine Institute (SVI) had delivered more than 640 doses of its experimental cAd3-Marburg vaccine to Ethiopia. This initiative is being implemented in close collaboration with the Ethiopian MoH, the United States Department of Health and Human Service through its Administration for Strategic Preparedness and Response (ASPR) and the Biomedical Advanced Research and Development Authority (BARDA), AHRI, and other international public health partners [15]. The Marburg vaccine candidate deployed in Ethiopia in late 2025 (cAd3-Marburg) was developed by the SVI, with early development initiated in collaboration with the U.S. National Institutes of Health (NIH) and Okairos (a biotech company later acquired by GlaxoSmithKline (GSK) in 2013). In 2019, the SVI entered an exclusive agreement with GSK to advance the development of this Marburg prophylactic vaccine. The development and manufacturing of Sabin’s cAd3-Marburg vaccine is funded by the BARDA, part of the U.S. Department of Health and Human Services. The vaccine is based on GSK’s Chimpanzee Adenovirus Type 3 (cAd3) platform [17]. The vaccine administration was set to begin on 08 December 2025. Sabin and the Ethiopian MoH have entered into a clinical trial agreement under which Sabin is providing BARDA-funded investigational cAd3-Marburg Vaccine doses for a two-cohort Phase 2, rapid response, open-label, randomized trial to assess safety, efficacy, and immunogenicity in selected areas of southern Ethiopia and the Sidama region [18]. The trial is a Phase 2, open-label, randomized study conducted in two cohorts and led by the AHRI together with the MoH. It evaluates a single-dose intramuscular vaccine. The vaccine is administered as pre-exposure prophylaxis for frontline healthcare workers and other individuals at high risk. In addition, it is used in a ring vaccination strategy for people who have been in contact with confirmed cases. Participants are grouped into two cohorts: Cohort A: Includes high-risk health professionals and individuals with direct contact with confirmed cases within the past 21 days and Cohort B: Includes other eligible healthcare workers and contacts [17]. The supplied vaccine doses are currently being utilized in a Phase II clinical trial (ClinicalTrials.gov ID: NCT06620003) targeting populations at elevated risk, including healthcare workers, frontline responders, and individuals who have been in contact with confirmed cases. In addition, ASPR has announced its commitment to provide up to 2,500 additional doses of the investigational vaccine to Ethiopia as part of the ongoing response to the outbreak, further strengthening preparedness and protection efforts among high-risk groups. This vaccine has also been used in Rwanda and other African sites (Uganda, Kenya) in Phase 2 trials (ClinicalTrials.gov ID: NCT05817422). The findings indicate that the earlier Phase 1 trials demonstrated safety and immune response generation in humans, but efficacy in preventing MVD in outbreaks is still being studied [19]. Additionally, the Global Clinical Trial Efforts demonstrated that multiple Phase 2 trials are underway in Uganda, Kenya, and Ethiopia. These studies aim to assess safety, immunogenicity, and effectiveness in outbreak settings [18].

Despite the absence of approved therapies, investigational medical countermeasures have been deployed on an emergency basis to support post-exposure prophylaxis (PEP) and patient treatment. As of 3 December 2025, Ethiopia had received 1,200 doses of remdesivir, an antiviral agent with supportive preclinical evidence against filoviruses, provided by Gilead Sciences under an emergency use authorization granted by the Ethiopian Food and Drug Authority. Remdesivir as a treatment regimen was administrated as soon as possible after diagnosis as a 200 mg intravenous (IV) infusion as a loading dose, followed by a maintenance dose of 100 mg IV infusion every 24 hours for a duration of treatment with 10 days minimum, but extends to 14 days, if the patients has not had a negative RT-PCR test in 10 days. It was also used as a short-course PEP for asymptomatic healthcare workers and other high-risk contacts who had been exposed to a patient with MVD without appropriate PPE within the previous 10 days. The aim was to reduce disease severity and potentially prolong the incubation period. The same loading and maintenance doses were used; however, the treatment duration was limited to three days [7].

In addition, on 4 December 2025, the ASPR announced the allocation of up to 25, small number of treatment courses of Mapp Biopharmaceutical’s investigational monoclonal antibody, cutting-edge therapeutics, MBP091, to Ethiopia. Of these, five treatment courses were delivered to the country on 5 December 2025 [18]. The investigational monoclonal antibody therapy MBP091 is administered as a single IV infusion at a dose of 100 mg/kg body weight, typically delivered over a period of approximately two hours [7,15]. Regrading the utility of this approach in other Africa countries such as in Rwanda’s 2024 outbreak, Gilead Sciences, in partnership with Africa CDC and Rwanda’s MoH, donated 5,100 vials of remdesivir to support Rwanda’s response to the MVD. This emergency donation aims to provide treatment to those affected by the virus following negotiations led by Africa CDC. However, remdesivir alone or in combination with monoclonal antibody treatment (MBP091) complemented with supportive care has improved the clinical outcomes of patients. Several experimental therapies are currently under investigation, including antiviral drugs such as favipiravir, galidesivir, obeldesivir, and remdesivir, along with monoclonal and polyclonal antibodies (e.g., polyclonal IgG, monoclonal antibody MR-78-N; MR82-N; MR191-N; monoclonal antibodies MR186-YTE and MBP091). Furthermore, substantial progress is being made in vaccine development, with promising candidates including adenovirus-vectored vaccines, DNA vaccines, and the recombinant vesicular stomatitis virus (rVSV) vaccine. Moreover, innovative preventive and treatment strategies-such as synthetic hormones like estradiol benzoate, small interfering RNA (siRNA), interferon-β therapy, and phosphorodiamidate morpholino oligomers-are emerging as potential options for MVD management [20].

## Conclusion

The confirmation of MVD in Ethiopia in November 2025 marks a serious clinical and public-health event with significant regional implications. Even so, national authorities have acted swiftly, and international partners are already providing support. Because the MARV is highly fatal and lacks any approved treatment or vaccine, prevention and containment remain the most effective tools for limiting its impact. Early developments—nine confirmed cases, several deaths, and a rapid, coordinated multi-agency response demonstrate encouraging levels of detection, transparency, and readiness. However, the current cluster could still grow, particularly given the risks associated with cross-border movement and the ecological conditions that favor zoonotic transmission. Immediate priorities include rigorous contact tracing, strong IPC practices, and implementation of robust public health strategies such as risk communication and community engagement (RCCE), which play a crucial role in raising awareness, promoting behavioral change, and encouraging communities to adopt preventive measures, and dispelling rumors and mis/disinformation from digital social media outlets. Close collaboration between local and global partners, transparent data sharing with the neighboring countries such as Kenya, South Sudan, Uganda (including genomic sequencing results), and targeted support to strengthen local clinical capacity will be vital in containing the outbreak and preventing further spread. Sustained monitoring and rapid operational research to address existing knowledge gaps will also be essential to curb transmission and protect communities in Ethiopia and neighboring countries.

## Recommendation

In light of the recent MVD event and the ongoing lessons from other African countries, Ethiopia has a critical opportunity to strengthen public health preparedness and build a more resilient health system. The following recommendations are proposed to enhance early detection, coordinated response, and long-term system readiness.

- Strengthening surveillance and early detection systems through both community-based and facility-level surveillance mechanisms and integrating MVD alerts into existing digital surveillance platforms.
- Enhancing RCCE via local media, community influencers, religious leaders, and health extension workers to improve trust and support rapid dissemination of lifesaving information.
- Strengthening IPC capacities, ensuring the availability of PPE, and training frontline health workers on safe case management.
- Bolstering cross-border and regional collaboration.
- Strengthening One Health coordination for zoonotic threats and ensuring availability mental health and psychosocial support during outbreaks at the epicenter and adjacent districts for handling fear, anxiety, and stigma.

Healthcare workers must maintain a high level of vigilance when identifying suspected cases of MVD. Safe and dignified burials must follow strict IPC protocols [21], with only trained health personnel handling the bodies. At the same time, cultural and religious practices should be respected as much as possible during burial services. Adhering to established guidelines [7,21] is essential for distinguishing MVD from other acute febrile illnesses (AFIs), many of which can appear very similar in their early stages. Given that the incubation period for MVD can range from 2 to 21 days, careful clinical assessment and strict adherence to case-definition criteria are vital. This helps prevent misclassification, supports early detection, and enables a rapid and effective public health response. Finally, the MoH should hold regular debriefing sessions to share updated information and clarify event-related details with the public. Doing so will help counter misinformation and strengthen public trust.
